# GIST and Ghrelin: To Be or Not to Be?

**DOI:** 10.3390/diagnostics11081361

**Published:** 2021-07-29

**Authors:** Irene Alexandra Spiridon, Delia Gabriela Apostol Ciobanu, Simona Eliza Giușcă, Dan Ferariu, Iulia Cătălina Pleşca, Irina Draga Căruntu

**Affiliations:** 1 Department of Morpho-Functional Sciences I—Morphopathology, “Grigore T. Popa” University of Medicine and Pharmacy, Strada Universității 16, 700115 Iași, Romania; simona-eliza.giusca@umfiasi.ro; 2 Department of Pathology, Regional Institute of Oncology, Str. General Henri Mathias Berthelot 2-4, 700483 Iași, Romania; d_ferariu@yahoo.com; 4 Department of Morpho-Functional Sciences I—Histology, “Grigore T. Popa” University of Medicine and Pharmacy, Strada Universității 16, 700115 Iași, Romania; irina.caruntu@umfiasi.ro; 3 Science Research Department, Institute of Interdisciplinary Research, “Alexandru Ioan Cuza” University, Strada Lascăr Catargi 54, 700107 Iași, Romania; iulia.plesca@uaic.ro

**Keywords:** ghrelin, GIST, carcinogenesis, pathology, prognosis

## Abstract

Background: Ghrelin is the orexigenic hormone secreted mainly by the stomach. Its involvement in neoplastic development has been studied in gastrointestinal adenocarcinomas. Our paper aims to evaluate the influence of the ghrelin axis in gastrointestinal stromal tumors (GISTs). Materials and Methods: The study design included two groups of patients, 46 with gastric GISTs and 30 with obesity. Archived tissue samples were evaluated for the presence of gastritis and *H. pylori*. Immunohistochemical expression of ghrelin and its receptor (GHS-R) was assessed. Results: All GISTs showed absent immunohistochemical expression for ghrelin, while GHS-R displayed a particular pattern, with notable differences in intensity (*p* = 0.0256) and percentage of stained cells (*p* < 0.00001) in the periphery vs. core of tumors. Positive ghrelin expression was lower in the gastric mucosa of the first group compared to the second group (*p* < 0.001). Conclusion: The ghrelin axis can influence GISTs carcinogenesis through activation of GHS-R. A previously described direct autocrine/paracrine mechanism is not supported by our findings.

## 1. Introduction

Gastrointestinal stromal tumors (GISTs), although rare, are the most common mesenchymal malignancies of the gastrointestinal tract (GIT) [1], accounting for a variable incidence around the globe, estimated at 7—15/106/year according to recent population-based studies [2,3,4]. Their point of origin can be traced back to the interstitial cells of Cajal (ICC), pacemaker cells located within the muscle layers [5,6,7].

While most GISTs arise in the stomach, a smaller number of tumors develop in the small bowel [1,8] and in other locations (esophagus, colon, rectum) [9,10,11]. Interestingly, these tumors can also occur outside of the GIT, in rare sites such as the gallbladder, omentum, mesentery, and retroperitoneal space, and are commonly referred to as extragastrointestinal stromal tumors (EGIST) [12,13].

GIST carcinogenesis revolves around c-KIT gain-of-function mutations [14], located on exons 11 and 9 or PDGFR-α mutation, on exons 12, 14, and 18 [15,16]. While these are the most frequently detected mutations, other alterations in genotype have also been identified [17,18,19,20]. Immunophenotypically, GISTs share a common c-KIT and CD34 immunohistochemical (IHC) expression with ICC, and it has been demonstrated that a gain-of-function KIT mutation in these cells leads to a precursor lesion known as ICC hyperplasia [21,22,23]. These observations show that an appropriate context is needed for a particular KIT mutation to have transforming activity.

GISTs exhibit a spectrum of biological behaviors, ranging from incidental, slow growing/stationary neoplastic proliferations to highly aggressive tumors, with widespread metastatic potential [24]. Hence, the need for a risk stratification system became imperative and was first developed in 2002 [25] and later refined in 2006 [16]. Gastric GISTs typically show a lower malignant potential than tumors arising in non-gastric sites [26], but the circumstances leading to these different profiles of aggressiveness remain unknown.

Ghrelin is the ligand of the growth hormone secretagogue receptor [27] and has both peripheral and central action [28], with a wide array of effects in both physiological and pathological settings [28,29]. Its role is not only linked to energy metabolism and appetite control [30,31,32] but also inflammation and oxidative stress [33,34], the modulation of cardiac activity and post-injury myocardial changes [35,36], stress and anxiety [37,38], anorexia and other psychiatric conditions [39,40], neurodegenerative disorders [41] and sepsis [42,43]. The longstanding contribution of ghrelin to carcinogenesis and tumor progression, especially in GIT malignancies [44,45] has been demonstrated through positive IHC and mRNA expression in endocrine and non-endocrine tumors [46,47] spanning across a multitude of organs and systems [45].

Ghrelin is produced by the oxyntic region of the gastric mucosa, where X/A-cells rarely synthesize any other hormone or hormone-like peptide [27,28,29]. These ghrelin-producing cells (GhrC) are closed cells, in intimate contact with parietal cells and with the direct discharge of their secretion into the local vasculature, without luminal release [48,49,50,51]. The sex of the patients has a significant contribution to both distribution of GhrC and peripheral concentration, with plasma levels of ghrelin being higher in females than in males [52,53,54]. The physiological actions of this hormone are modulated through the interaction with its receptor (GHS-R), which currently has two identified splice variants, GHS-R 1a and GHS-R 1b, widely expressed in both normal and tumor tissue [55,56,57]. While the specifics of this interaction have been intensely investigated and revolve mainly around the enzymatic acylation of ghrelin through ghrelin-o-acyltransferase [58], recent evidence suggests new intricacies in both circulating and local tissue interactions, with novel splice variants of ghrelin identified and investigated as potential promotors of tumor progression and invasion [59,60,61].

The involvement of the ghrelin axis in GISTs has been reported in only one study, demonstrating a potential link through IHC and molecular expression [62]. The authors reported positive ghrelin and GHS-R expression in GISTs but found no statistical correlation with clinicopathological parameters. Assumptions regarding the existence of an autocrine/paracrine loop and other potential mechanisms behind ghrelin-linked tumor progression were only more recently advanced [63]. However, the current state of the art demands more research in order to discern the link between ghrelin and GIST tumorigenesis and prognosis.

In this context, we aimed to analyze the presence of ghrelin and its receptors in gastric GISTs and compare it with the expression of the ghrelin and its receptor in the stomach of patients with obesity, as a non-neoplastic pathology, in order to outline these partially characterized patterns of expression.

## 2. Materials and Methods

### 2.1. Subjects

The study included two groups: (i) 46 patients with gastric GISTs and (ii) 30 patients with obesity that underwent laparoscopic sleeve gastrectomy (LSG). All cases were retrospectively selected from the Gastroenterology Unit and Surgical Units of “Sf. Spiridon” Emergency County Hospital Iasi and the Regional Institute of Oncology Iasi.

The cases in the first group were diagnosed as GIST and confirmed through histopathological and IHC examination using a panel of markers (CD117, CD34, DOG1). Data concerning the molecular profile of these tumors were not available. For the second group, the surgical specimens from LSG presenting with peptic ulcer, atrophic body gastritis with possible autoimmune etiology, or other types of local or systemic neoplastic lesions were excluded. Thus, we avoided the overlap with lesions specific to these conditions, including the possible effects of associated therapy on the gastric mucosa.

The Ethics Committees of the University and of both medical units approved the study protocol, based on the written informed consent of the patients.

### 2.2. Histological and IHC Examination

The tissue samples were fixed in 10% formaldehyde and embedded in paraffin, and 4–5 μm thick sections from each specimen were cut for histochemical stains and IHC examination. Standard hematoxylin-eosin for histological assessment and Giemsa for evaluation of *H. pylori* infection was performed. For each case, the gastric mucosa was examined and scored according to the updated Sydney system [36] to indicate the degree of inflammation, atrophy, intestinal metaplasia, and *H. pylori* density.

Ghrelin and *H. pylori* immunoreactivity were determined for both groups, while GHS-R expression was analyzed only in the first one. The slides were pretreated with Epitope Retrieval Solution (pH 9, 96oC, 20 min) and incubated overnight with the primary antibodies (anti-ghrelin monoclonal antibody, ab209790, Cambridge, MA, USA; anti-GHR-S polyclonal antibody ab85104, Cambridge, MA, USA; anti-*H. pylori* antibody, EP279, CellMarque, Rocklin, CA, USA) diluted 1:5000, 1:250 and 1:150, respectively. Diaminobenzidine–hydrogen peroxide was used as chromogen, and the sections were counterstained with diluted hematoxylin. Positive external controls included samples of the gastric mucosa (oxyntic area) with *H. pylori*-associated gastritis. For negative controls, the primary antibody was omitted and replaced with normal serum at equivalent concentration.

### 2.3. Quantitative and Topographic Evaluation

Ghrelin expression was evaluated in the group with gastric GISTs within the tumor, both in the periphery and the core, and in the overlying mucosa, while in the second group its expression was assessed within the gastric mucosa. GhrC density was evaluated in 10 consecutive high-power fields (HPF) at ×400 magnification. The results were expressed in the number of GhrC/10 HPF [64].

GHS-R expression was evaluated only in gastric GISTs, within the core of the tumors, and at the invasive front in 10 consecutive HPF. For each of the two areas examined, the immunostaining intensity in the nucleus and cytoplasm was noted as follows: 0—negative; 1—weakly positive; 2—moderately positive; 3—intensely positive. The number of positive cells was expressed as a percentage of positive cells, using 10% intervals.

Immunoreactivity for *H. pylori* was reported as positive or negative.

### 2.4. Statistical Analysis

The statistical analysis was conducted by coding in Python programming language version 3 (Python Software Foundation, Wilmington, DE, USA). The computational environment used was Jupyter Notebook version 6.1.4 (Austin, TX, USA) with Anaconda Navigator version 1.10.0 (Austin, TX, USA). Several libraries were used: pandas (version 0.24.2), numpy (version 1.16.2), matplotlib (3.0.3), seaborn (version 0.9.0), scipy (version 1.2.1) together with the statsmodel package (version 0.9.0) for both the computations and the creation of graphs. The results from the quantitative analysis are shown as percentiles and/or mean ± SD. The test used for comparisons were Fisher tests for categorical comparisons and the Student’s *t*-test for means comparison. The Mann–Whitney U test was used to compare continuous versus categorical variables. The relationships between GhrC and other morphological variables were examined by linear regression and Spearman correlation coefficient analyses. A *p*-value < 0.05 was considered to be statistically significant.

## 3. Results

### 3.1. Clinicopathological Characteristics

The first group included 46 patients diagnosed with gastric GIST, 47.8% (22) males and 52.2% (24) females (average BMI < 40). The average age was 65.7 years (ranging from 39 to 91 years old). On histopathological examination, the most prevalent GIST subtype was spindle cell (50%), followed by the mixed subtype, with 43.48%, and epithelioid subtype, with 6.52%. Most tumors were diagnosed as pT2 (41.3%) and pT3 (34.78%), with a low to moderate degree of pleomorphism (82.6%) and only 17.4% with high pleomorphism. Mitotic activity was evaluated as the number of mitosis/50HPF and recorded as mostly low, with 58.7% of tumors having >5 mitoses/50HPF, while 41.3% had less than 5 mitoses/50HPF. These parameters are summarized in Table 1. The IHC profile of gastric GISTs showed positivity in the majority of cases for CD117 and DOG1 (89.13%), and the number of CD34 positive tumors was slightly smaller (84.78%).

The second group was composed of 30 patients diagnosed with morbid obesity (average BMI > 40), treated by LSG. There were 33.33% (10) males and 66.67% (20) females, with an average age of 44.83 years (ranging from 20 to 76 years old).

### 3.2. Ghrelin and GHS-R in Gastric GIST

**Figure 1:**
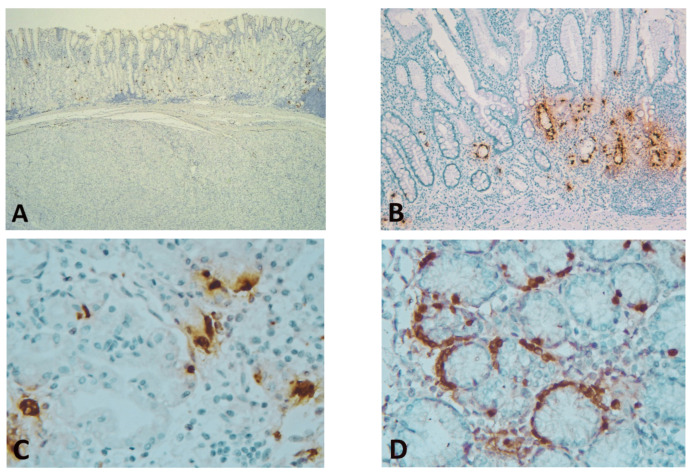
(**A**) Spindle cell GIST showing complete negativity for ghrelin (positive ghrelin-producing cells (GhrC) in overlying gastric oxyntic mucosa—internal control) (immunohistochemical (IHC), Anti-ghrelin antibody, ×25 magnification); (**B**) Gastric mucosa (corpus) of laparoscopic sleeve gastrectomy (LSG) patients with associated chronic gastritis and intestinal metaplasia, showing predominant lower 2/3 disposition of GhrC (IHC, Anti-ghrelin antibody, ×100 magnification); (**C**) Single cells or disposition in small groups of GhrC with intense cytoplasmic staining in the oxyntic mucosa of LSG patients; the cell count was performed at this magnification (IHC, Anti-ghrelin antibody, ×400 magnification); (**D**) Intense cytoplasmic staining of GhrC showing linear hyperplasia in isolated cases of LSG patients (IHC, Anti-ghrelin antibody, ×400 magnification).

Immunostaining of GISTs for ghrelin showed complete negativity (with positive internal and external control) (Figure 1).

**Figure 2:**
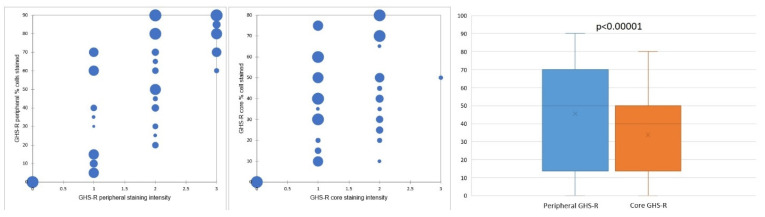
GHS-R staining distribution and preferential staining pattern in tumor periphery vs. tumor core.

Concerning the percentage of positive cells, gastric GISTs stained positively for GHS-R in most tumor samples (40 cases—86.95%). All these cases displayed a fine granular (occasionally coarse) cytoplasmic staining pattern, while a nuclear/perinuclear staining pattern was observed in only 38 cases (82.61%). Therefore, 8 cases (17.39%) lacking cytoplasmic and/or nuclear/perinuclear immunoreaction were considered negative for GHS-R. The evaluation of the nuclear/perinuclear staining pattern revealed that 27 out of 38 GHS-R positive samples (76.31%) displayed extensive staining in the periphery compared to the core of the tumor (Figure 2). This aspect was considered a preferential distribution pattern. The quantitative assessment showed that in the periphery of the tumors, 8 cases (representing 21.05%) had more than 80% positive cells, while in the core, no tumor was displaying more than 80% positive cells. On the other hand, 18 cases (47.36%) had positive GHR-S immunoreaction in less than 50% of peripheral tumor cells, whereas 32 cases (84.21%) presented a similar percentage of positive cells in the core. Only 5 cases (13.15% of tumors) displayed the same percentage of stained cells in the core and periphery and 4 cases (10.52%) showed more immunoreactive cells within the tumor core. The statistical analysis confirmed significant differences between the overall percentage of GHS-R positive tumor cells in the periphery vs. the core (*p* < 0.00001).

**Figure 3:**
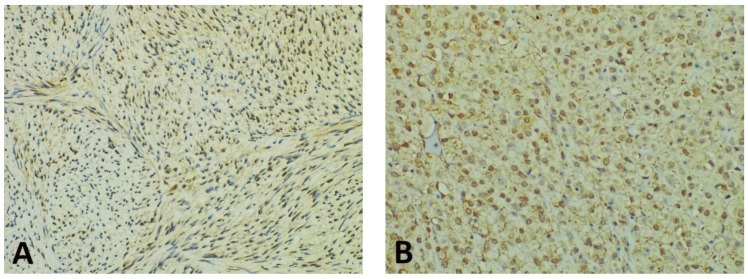

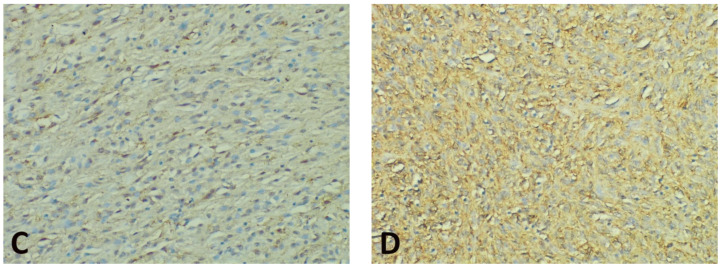
Representative photomicrographs showing GHS-R IHC staining in GISTs, with examples of the evaluation method previously described. Cytoplasmic immunoreactivity was observed in most cases, while a preferential staining pattern was noted in tumor periphery vs. core (IHC, anti-GHS-R antibody, ×200 magnification). (**A**) Nuclear immunoreactivity in spindle GIST, score 3 (intense); (**B**) Nuclear immunoreactivity in epithelioid GIST, score 2 (moderate); (**C**) Nuclear immunoreactivity in spindle GIST, score 1 (weak); (**D**) Absent nuclear immunoreactivity in spindle GIST (score 0).

Concerning the intensity of the nuclear/perinuclear stain, scored from 0 to 3, comparable differences were also noted between the tumor periphery and core (Figure 3). The highest intensity score (with a value of 3) was observed in 8 cases (21.05%) in the tumor periphery, and only in one case (2.63%) within the tumor core. An intensity score of 2 was assessed in 21 cases (63.15%) in the periphery vs. 16 cases (42.10%) in the tumor core. On the contrary, the lowest intensity score (with a value of 1) was present in only 9 cases (23.68%) at the invasive front and in 21 cases (55.26%) at the tumor center. Following the integrated analysis of the staining intensity, our data indicated that approximately half of the tumors (20 cases—52.63%) had the same level of staining intensity, the other 18 cases (47.36%) presenting higher immunoreactivity in the periphery vs. tumor core. The statistical analysis also confirmed significant differences between the staining intensity in the two tumor areas (*p* = 0.0256).

The statistical analysis aimed at the correlation of the tissue expression of GHS-R (assessed as a percentage of positive cells) and clinicopathological parameters indicated only weakly significant differences between male and female patients (*p* = 0.035). No correlations with tumor size, tumor prognostic group, or mitotic index were present.

### 3.3. Quantitative Assessment and Topographic Distribution of GhrC in Gastric Mucosa

In the first group, GhrC evaluation showed an average of 172.52 ± 106 cells/10 HPF, with immunoreactive cells showing a cytoplasmic, intensely positive granular staining. GhrC were either isolated or in pairs or groups no larger than 6 cells, typically located in the lower 2/3 of the gastric mucosa (56.52%) or limited exclusively to the lower 1/3 of the mucosa (32.60%). In the antropyloric region, GhrC showed a basal distribution, in the lower third of the mucosa (77.77%), with an average of 60.4 ± 33.15 cells/10 HPF, while in the body, the distribution was mostly in the lower 2/3 of the mucosa (69.44%), with an average of 203.66 ± 97.85 cells/10 HPF. Few cases displayed a haphazard distribution of ghrelin-positive cells (10.87%).

Chronic gastritis was present in the overlying mucosa in 84.78% of patients, with only 23.07% of cases showing signs of active disease (neutrophilic infiltrate). Variable degrees of atrophy were present in 41.3% of patients, and only 19.56% showed intestinal metaplasia. The presence of *H. pylori* was confirmed by Giemsa and antibody stain in 32.60% of cases (Table 2). Expression of GhrC was weakly correlated with the presence of *H. pylori* (*p* = 0.046154), but independent of other factors like mononuclear inflammatory infiltrate, atrophy, or metaplasia.

In the second group, the IHC examination revealed an average of 268.9 ± 134.53 cells/10 HPF, predominantly located in lower 2/3 of the gastric mucosa (83.33%), with a similar pattern of expression in secreting cells. In three cases, GhrC hyperplasia (both nodular and linear) was observed (Figure 1). Chronic gastritis was present in 70% of analyzed cases, with only 10% of them associating active gastritis. Atrophy was noted in 33.33% of patients, and lesions of metaplasia, either complete or incomplete, were observed in 16.66% of cases. *H. pylori* colonization of superficial mucosa and gastric pits was detected in 30% of cases (Table 2). While GhrC expression was not associated with *H. pylori* infection, it correlated with the presence of metaplasia (*p* = 0.043).

The density of GhrC was higher in the oxyntic mucosa of *H. pylori*-negative patients than in *H. pylori*-positive patients of both groups (Table 3). There were no significant differences between male/female patients and the total number of GhrC.

**Figure 4:**
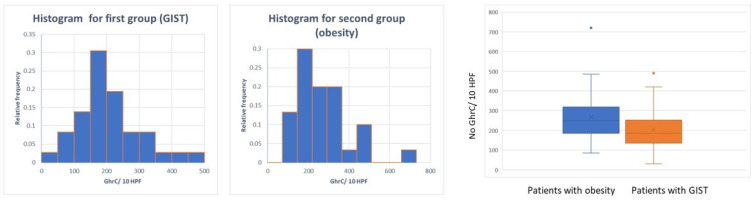
Distribution of GhrC in overlying mucosa of gastric GISTs vs. obese patients.

The comparison between the two groups in terms of GhrC expression in the gastric mucosa showed a significantly lower number of GhrC in the overlying gastric mucosa of GISTs, with a mean value of 172.52 cells ± 106 cells/10 HPF as compared to the group of patients with obesity with 268.9 ± 134.53 cells/10 HPF (*p* < 0.001) (Figure 4).

## 4. Discussion

The search for novel prognostic markers and additional mechanisms involved in GISTs is ongoing, with attention focusing on tumor microenvironment or local endocrine regulation. However, little is known about the role that ghrelin could play in the GISTs’ progression. A systematic literature search regarding the expression and influence of this hormone and its receptor in GISTs renders scarce data, with only one report characterizing the expression of the ghrelin axis through IHC and mRNA assays [62].

Our paper illustrates the IHC expression profile of the ghrelin and its receptor in gastric GISTs, while also comparing the variable localization and pattern of expression of GhrC in the overlying mucosa of tumors with the expression in the mucosa of patients with obesity who have undergone LSG. Obesity is a non-neoplastic disorder with major metabolic alterations and a significant contribution to the development of some epithelial malignancies [65]. In our study design, we have opted for the use of LSG specimens as a comparison group in order to investigate ghrelin expression based on the advantage of analyzing full-thickness gastric mucosa, being aware at the same time that this pathological state is considered to be unassociated with GISTs (2). In spite of plasma ghrelin levels being decreased in patients with obesity [66,67], the current body of knowledge reports similar levels of expression of GhrC in obese vs. non-obese control patients [68,69,70,71].

The one study that has examined the expression of ghrelin and its receptors in GISTs was conducted on a small group—only 22 cases [62]. mRNA expression was determined in 6 cases and compared to a control group represented by 5 endocrine pancreatic tumors, with mean ghrelin mRNA levels in GISTs detected at similar levels to those in the control group [62]. IHC expression was reported in 17 out of the 22 examined tumors, with variable levels of positivity [62]. However, no statistical correlations to important clinicopathological factors such as tumor location, tumor size, mitotic activity, or risk of recurrence were obtained [62]. The proposed explanation for these results was the small study sample hampering the acquisition of relevant data [62,63]. It has been shown that GISTs also have a neuroendocrine phenotype, expressing both peptide hormones and receptors [72]. The existence of a ghrelin autocrine/paracrine loop in GISTs was suggested, indicating a potential role that the ghrelin axis might play in tumor development and progression [62,63]. However, the current state-of-the-art regarding ghrelin expression in GISTs does not include sufficient evidence to confirm the existing hypotheses on its involvement in carcinogenesis, and the topic is still a matter of debate.

In contrast to this single report [62], our analysis of the IHC expression of ghrelin rendered all negative results in the tumor samples, with positive and negative controls on all slides. We emphasize the use of a monoclonal anti-ghrelin antibody, with better specificity than the polyclonal one [62], arguing for the absence of ghrelin protein expression within the tumor.

Ghrelin has shown implication in other pathologies through the activation of the PI3K/AKT/mTOR pathway [73,74], a pathway also investigated in GISTs for its potential therapeutic role [63]. Our study contradicts the possibility of a ghrelin autocrine/paracrine mechanism employed by these tumors and refutes the use of ghrelin protein expression as a potential biomarker. A possible reason for this discrepancy in results is detailed below.

The existence of several peripheral forms of ghrelin as a result of splicing shows the complexity of this hormonal interaction [60], and studies reporting positive In1-ghrelin expression in neuroendocrine tumors compared with native ghrelin expression with the help of quantitative PCR assay strongly point to the potential role of In1-ghrelin as a prognostic marker [61]. These recent advances can anticipate the translation in study designs from gene expression to protein levels evaluated through the help of IHC, a more accessible technique in pathology laboratories.

However, the contribution of circulating ghrelin levels in tumorigenesis and neoplastic progression cannot be entirely rejected. The GhrC profile in overlaying mucosa of GISTs and the GHS-R presence in the tumor are solid arguments to sustain this assumption. To this end, our results showed a statistically significant decreased GhrC expression in this territory when compared to the group of patients that underwent LSG. As mentioned before, the number of GhrC in the gastric mucosa of obese patients was similar to that of non-obese controls [69] and was influenced by the inflammatory status and the presence of *H. pylori*-associated gastritis [69,75,76]. Therefore, we considered the LSG specimens as a valuable non-tumor control group. In our opinion, the decrease of GhrCs in GISTs’ overlying mucosa in the absence of a paracrine negative feedback loop could indicate the modulation of the ghrelin axis expression through other secretory factors released by these tumors. While being aware that GhrC releases the secretion directly into the bloodstream [52], their local effect is considered minimal and it can be hypothesized that the interaction between ghrelin and its receptor is modulated by the circulating levels. As a result of the variations in ghrelin plasma levels, it can be seized by the cells that have GHS-R.

For GHS-R expression, our results were once again different from those reported by Ekeblad et al. [62]. Moreover, our work demonstrated that IHC GHS-R expression was positive in both tumor core and invasive front, with statistically significant differences between these territories in both intensity and percentage of positive tumor cells. We specify the possible utility of developing a scoring system based on the percentage of positive cells and staining intensity, a tool currently absent in the literature. The refining of such a system on a larger number of GISTs with various localizations could prompt correlations with the clinicopathological data and prove a useful tool in the microscopic assessment of these tumors. While the tumor expression of ghrelin was null, the GHS-R expression should be regarded as solid evidence for the involvement of ghrelin in modulating tumor behavior.

Nonetheless, the variation in phenotype and protein expression at the level of the invasive front can bring a wide array of information with an impact on patient prognosis. This is a hot topic for tumors of epithelial origin such as squamous cell carcinoma [77,78,79] or adenocarcinomas of the digestive tract [80], but few studies focus on non-epithelial tumors, and no data are reported for GISTs. Hence, this overtly different pattern of GHS-R expression, with marked accentuation at the periphery, could also justify the timeline spanning from resection, if incomplete, to tumor recurrence.

It is worth noting that the interaction between ghrelin and its receptor has been studied in few malignancies. Published data point out their involvement in invasiveness and migration of tumor cells in the case of pancreatic and gastric adenocarcinoma [81,82], through the activation of the PI3-K/Akt/mTOR pathway [81,83]. In the case of glioma cells, NF-kB activation can contribute to ghrelin-induced cell migration [84]. Likewise, ghrelin inhibition significantly blocked the migration and invasion of human colon cancer cell lines [85].

Some authors propose that the expression status of GHS-R represents the response to the ghrelin-GHS-R activation system during alterations in hormone homeostasis in the context of cancer cachexia [86]. However, cachexia is not a dominant element in the evolution of a GIST patient, showing a low incidence in patients with this type of malignancy [22,62].

A link between *H. pylori* infection and ICC has been recently reported in the context of delayed gastric motility disorders, the presence of the pathogen resulting in a decrease in the number of pacemaker cells in gastric tissue [87]. Although this was not part of the common clinical setting of GISTs patients, almost a third of them had associated *H. pylori* infection, with no statistical correlation to the tumors. The impact of *H. pylori* on the gastric microenvironment may lead to alterations and a possible impact on both neoplastic development and a difference in prognosis for gastric GISTs. This concept was recently explored in one study on a multi-ethnic group of 71 patients and revealed a strong association between *H. pylori* infection and GIST [88]. However, our data show no correlation between the presence of the pathogen and the clinicopathological factors, provided that a larger group and other detection methods could be employed to clarify these aspects.

Between hypothesis and conflicting results, the definitive role of ghrelin in the mechanism of GIST-specific carcinogenesis is not yet deciphered. More studies are needed, with a comprehensive and methodical approach to analyze circulating serum levels of ghrelin splicing variants, tissue protein expression and link them to the tumor genetic profile and clinicopathological factors. Following this reasoning, our data represent a valuable contribution and a further step in understanding the relationship between enteroendocrine cells and GISTs, with results that have the potential to translate into prognostic assessment and targeted therapy.

## Figures and Tables

**Table 1:** Clinicopathological characteristics of gastric Gastrointestinal stromal tumors (GISTs).

Age	65.7 years old
Gender (Male/Female)	22/24 cases
Tumor size (cm)	
≤ 2	2 cases (4.34%)
>2 and ≤5	19 cases (41.30%)
>5 and ≤10	16 cases (34.78%)
>10	9 cases (19.56%)
Mitotic index (no. mitosis/50 HPF)	
<5	27 cases (48.69%)
>5	19 cases (41.30%)
Histological subtype	
Spindle type	23 cases (50%)
Epithelioid type	3 cases (6.52%)
Mixed type	20 cases (43.47%)
Tumor necrosis	
Yes	18 cases (39.13%)
No	28 cases (60.87%)
Risk classification	
Very low	15 cases (32.60%)
Low	8 cases (17.40%)
Intermediate	11 cases (23.91%)
High	12 cases (26.09%)

**Table 2:** Histological changes in the overlying mucosa of GISTs and in the mucosa of obese patients group.

Histopathological Changes	GIST Group—No. of Cases (%)	Obese Group—No. of Cases (%)
Normal mucosa	7 (15.21)	10 (21.73)
Gastritis	39 (84.78)	30 (65.21)
Active gastritis	9 (19.56)	3 (6.52)
Atrophy	24 (52.17)	10 (21.73)
Metaplasia	9 (19.56)	5 (10.86)
*H. pylori* positive	15 (32.60)	9 (19.56)
Total	46	30
Mean age (years)	65.7 years old	44.83 years old

**Table 3:** Mean density of GhrC (cells/10HPF) and* H. pylori* infection in gastric mucosa of patients with GIST and obese patients.

	GIST Group				Obese Group	
	Antral Mucosa		Oxyntic Mucosa		Oxyntic Mucosa	
*H. pylori* Infection	No.	Density	No.	Density	No.	Density
Positive	2	57 ± 35.35	13	241.69 ± 114.62	9	272.22 ± 183.06
Negative	8	61.25 ± 35.08	23	182.17 ± 82.04	21	267.47 ± 113.28
Total	10	60.4 ± 33.15	36	203.66 ± 97.85	30	268.9 ± 134.53

## Data Availability

All supporting data are provided in the current manuscript.
